# The Book World of Medicine and Science

**Published:** 1899-01-21

**Authors:** 


### THE BOOK WORLD OF MEDICINE AND SCIENCE.

GEORGE HARLEY, F.R.S. The Life of a London Physician. Edited by his daughter, Mrs. ALEC TWEEDIE. (London: The Scientific Press, Limited. 1899. Price 16s.)

SUCH a life as that of the late George Harley furnishes ample scope to the biographer, and when we add that in this case the biographer is the daughter of the man whose career is pourtrayed, that she deals with the subject sympathetically, that she was a participator in much which she describes, and that he was a man who not only wrote much for publication, but also left a large quantity of notes and jottings as to his thoughts and experiences, we need hardly say that, so far as material is concerned, Mrs. Alec Tweedie had everything to hand for the making of a most interesting story of a life. And in truth this is exactly what she has done. She has given in these pages a capital presentment of her father, of his life, of his character, of his work, and of the unique position which for so many years he occupied in London society; but she has done more, for she has given a series of pictures and a string of anecdotes which make the book interesting reading even to those who never knew Dr. Harley and have no particular concern with the scientific work with which he occupied himself, and by which he so quickly made himself a name. For from his early days till quite the end of his life Dr. Harley seemed to have had the fortune to attract or be attracted by curious and out-of-the-way people, leaders each in their own sphere, so that in this record of his life there are many strange touches dealing with subjects far outside what one might expect in the life of a successful London physician, which after all, when money has to be made and an establishment to be supported, is all too apt to become a very humdrum commonplace affair. The account of the time he spent in Paris gives an opening for various little stories, each of which will stand by itself. One about a pocket-knife that would pierce a five-franc piece is very well told, and his description of what he saw of Paris just after the *coup d'etat* although perhaps somewhat indicative of a desire to see adventures in everything is full of excitement. It is this constant succession of little tales that makes the volume an entertaining after-dinner book as well as a serious biography. "I had a friend," he says, "who, by some means or other, contrived to obtain pretty correct information concerning all events about to take place in Paris. On the evening before the distribution of orders and medals above alluded to, he called and startled me by asking if I should like to be present at the assassination of Louis Napoleon. On my inquiring when and where the event was to take place, the only reply he gave was 'Never you mind; if you want to see it, I'll take you.' . . . Where my friend got his information he would never deign to inform me, but subsequent events proved it to be well grounded," and then we get the story which we need not enter into any further. But if Dr. Harley found adventure in Paris his visit to Germany was even more exciting. His description of the last public decapitation which took place in Bavaria, is striking if gruesome. Certainly, if one reads Dr. Harley's description of what was done with a broad and heavy sword, and of the way in which by its means "in a second—aye, less than the twinkling of an eye—the sword swept like lightning though the neck, and the head was loose in the hand of the assistant," and compares it with Zola's description in his "Paris" of the operation of the modern guillotine, it does not seem that this instrument is as good as the old broad sword. The climax, however, of his adventures, was reached when in his endeavour to join Omar Pasha's army as a volunteer medico; he was caught by the Austrians, and condemned to be shot as a spy. Then we find him, his student days over, coming to settle down and make a home for himself in London. And here again we have a story of good luck, such as can have happened to but few of the aspirants for fame who flock to London in such ever-flowing streams, for he straightway obtained an appointment, and found himself at once on the ladder which later led to his assured position in the medical world of London. "A stranger in London, and without so much as even an acquaintance at University College, then the best teaching school in London, I acquired a paid appointment within five days of my arrival in the Metropolis." This was indeed good fortune. How he worked, how he wrote, how he gradually drew to himself consulting practice, always labouring at the scientific aspect of his profession, how he settled in Harley Street, married a wife, and became successful; these things are all written in this many-sided biography. And it is also written how in the midst of his success he was struck down by one of the greatest of misfortunes. Dependent as he was upon eyesight for everything, as physician, as writer, and as microscopist, he became blind. For nine months he remained in absolute darkness. Everything seemed to have been lost, but in the end he recovered, and soon his practice returned, and he took up again his old position. Tales of queer patients, of the curious doings in the Harley Street home, of the interesting people who were wont to meet there, and of the scientific hobbies with which Dr. Harley filled up the odd corners of a busy and ever active life, occupy a considerable portion of the book. As is stated upon the title-page, this is the life of a London physician. It is told, however, not as a mere chronology, but as a series of tableaux, by which the many-sided character of the subject is brought vividly before the reader. It certainly is a book to read.

MANUAL OF FIRST AID; BEING A TEXT-BOOK FOR AMBULANCE CLASSES AND A WORK OF REFERENCE FOR DOMESTIC AND GENERAL USE. By J. A. AUSTIN, M.D. (London: Sampson Low, Marston, and Co. 1898. Price 3s. 6d.)

THIS is a rather superior manual of first aid, something a little larger and more complete than the official manual, but running on much the same lines. In fact, as long as the examinations of the St. John Ambulance Association strictly follow the lines laid down in the book published under the auspices of the Association, no work on first aid is likely to step far out of the beaten track, which is, perhaps, a pity. As usual, we have the skeleton described, then the muscles and joints, and chapters on the blood, respiration, nervous system, digestion, &c., all of which, although matters full of interest to the average ambulance student, really give the ordinary man in the street but little help in deciding what to do in case of accident or how to give first aid. Still all knowledge is useful, although how the little pictures of blood as seen under the microscope assist in forwarding the particular object of the work before us may not be quite apparent. It has, however, become the fashion to teach all these things in ambulance classes, so we must not grumble at their being introduced into the manuals which are intended to be used by the students. All the practical part of the book is very well done, the whole work is well printed and illustrated, and will, we think, fully serve its purpose as a somewhat advanced manual of first aid. As a fact, however, those who attend the classes and go properly through the practical work ought to need very little help from any manual. Perhaps the second part of the title of the book best describes the position it is meant to occupy.

